# Allelic Variations of a Light Harvesting Chlorophyll A/B-Binding Protein Gene (*Lhcb1*) Associated with Agronomic Traits in Barley

**DOI:** 10.1371/journal.pone.0037573

**Published:** 2012-05-25

**Authors:** Yanshi Xia, Zhengxiang Ning, Guihua Bai, Ronghua Li, Guijun Yan, Kadambot H. M. Siddique, Michael Baum, Peiguo Guo

**Affiliations:** 1 International Crop Research Center for Stress Resistance, College of Life Sciences, Guangzhou University, Guangzhou, China; 2 College of Light Industry and Food Science, South China University of Technology, Guangzhou, China; 3 Hard Winter Wheat Genetics Research Unit, United States Department of Agriculture - Agricultural Research Service, Manhattan, Kansas, United States of America; 4 The Institute of Agriculture, The University of Western Australia, Crawley, Perth, Australia; 5 International Center for Agricultural Research in the Dry Areas, Aleppo, Syria; University of Hyderabad, India

## Abstract

Light-harvesting chlorophyll a/b-binding protein (LHCP) is one of the most abundant chloroplast proteins in plants. Its main function is to collect and transfer light energy to photosynthetic reaction centers. However, the roles of different LHCPs in light-harvesting antenna systems remain obscure. Exploration of nucleotide variation in the genes encoding LHCP can facilitate a better understanding of the functions of LHCP. In this study, nucleotide variations in *Lhcb1*, a LHCP gene in barley, were investigated across 292 barley accessions collected from 35 different countries using EcoTILLING technology, a variation of the Targeting Induced Local Lesions In Genomes (TILLING). A total of 23 nucleotide variations were detected including three insert/deletions (indels) and 20 single nucleotide polymorphisms (SNPs). Among them, 17 SNPs were in the coding region with nine missense changes. Two SNPs with missense changes are predicted to be deleterious to protein function. Seventeen SNP formed 31 distinguishable haplotypes in the barley collection. The levels of nucleotide diversity in the *Lhcb1* locus differed markedly with geographic origins and species of accessions. The accessions from Middle East Asia exhibited the highest nucleotide and haplotype diversity. *H. spontaneum* showed greater nucleotide diversity than *H. vulgare*. Five SNPs in *Lhcb1* were significantly associated with at least one of the six agronomic traits evaluated, namely plant height, spike length, number of grains per spike, thousand grain weight, flag leaf area and leaf color, and these SNPs may be used as potential markers for improvement of these barley traits.

## Introduction

Light-harvesting chlorophyll a/b-binding protein (LHCP) is one of the most abundant proteins of the chloroplast in plants. It roughly accounts for half amount of the chlorophyll involved in photosynthesis. The main function of LHCPs is collecting and transferring light energy to photosynthetic reaction centers [1]–[4]. Many homologous genes encoding LHCPs from various plant species belong to one of the 10 members in the gene family [5]–[7]. Four LHCPs of photosystem (PS) I, named LHCI, are encoded by the *Lhca1*, *Lhca2*, *Lhca3* and *Lhca4* [7]. Three major PS II associated LHCPs, designated as LHCII and encoded by *Lhcb1, Lhcb2 and Lhcb3*, are highly homologous and probably form homo- or heterotrimers [7], [8]. Three other PS II associated LHCPs have been designated as minor LHCPs, including inner antenna chlorophyll *a*-binding complexes CP29, CP26 and CP24 that are encoded by the *Lhcb4, Lhcb5 and Lhcb6* genes, respectively [8]. The minor LHCPs are monomeric and more closely associated with PS II than the major LHCPs [7], [9]. However, the roles of each LHCP in the structure, function and regulation of the light-harvesting antenna systems remain to be discovered [10].

Several studies have postulated that the LHCP genes were down-regulated in stress conditions such as cold [11], high-salinity [11], drought [12], [13] and infection by *Puccinia triticina* [14]. Moreover, a higher level of *LHCP* transcripts was detected in high osmotic adjustment (OA) plant of *Oryza sativa* spp. *japonica*, IR62266, than that in low OA CT9993 at a moderate level of dehydration stress [12]. Similarly, a higher level expression of a *LHCP* was observed in the drought-tolerant genotypes, Martin, than in the drought-sensitive genotype, Moroc9-75 under drought stress [13]. Ability of different accessions to adapt to stress conditions resides in their genetic diversity. Single nucleotide polymorphisms (SNPs) and small insertions and deletions (indels) are the most common forms of nucleotide variation in natural populations [15]. To date, the allelic variations in *LHCP* have not been systematically examined. The exploration of genetic variation in genes encoding LHCPs may facilitate a better understanding of functions of LHCPs and provide useful information and selection tools for plant breeders to improve plant with high photosynthesis efficiency.

Many techniques can be used for analysis of nucleotide variation within a gene. Sequencing is the most accurate approach, but is relatively expensive when applied in large numbers of individuals [16]. Since 2004, EcoTILLING, a variant of Targeting Induced Local Lesions in Genomes (TILLING) technique [17], has been increasingly used in several species for discovering nucleotide polymorphism of important genes in natural populations due to its high-throughput, accuracy, cost-effectiveness [18]–[20]. In sunflower, seven SNPs and two indels were identified in a *LHCP* region using EcoTILLING technology in 19 elite inbred lines [21]. In barley, allelic variations were identified in *mlo* and *Mla* resistance genes [22] and drought-related genes [23] using the same method.

In this study, a natural population of 292 barley accessions with diverse geographical origins was analyzed using EcoTILLING technology to examine allelic variation of an *Lhcb1* gene. A total of 23 nucleotide changes were detected with 31 distinguishable haplotypes in the germplasm collection. The potential association of SNPs with protein function changes was evaluated. Distribution of SNPs in accessions from different geographic origins (Africa, Middle East Asia, North East Asia, Arabian Peninsula, Australia and Europe) and genotypes (wild, cultivar and landrace) was investigated. In addition, association analysis between SNPs in the *Lhcb1* and six agronomic traits of barley has been performed.

## Material and Methods

### Plant materials and DNA extractions

A set of 292 barley (*Hordeum vulgare* L.) accessions was obtained from the International Center for Agricultural Research in the Dry Areas (ICARDA) (Table 1 and Table S1). These accessions contain 171 *H. vulgare* landraces (VUL-LA), 82 *H. vulgare* cultivars or improved genotypes (VUL-IG) and 39 wild relatives *H. spontaneum* (SPON), which were collected from 35 countries in six geographic regions including Africa, Middle East Asia, North East Asia, Arabian Peninsula, Australia and Europe.

**Table 1:** The geographic origins of the barley accessions used for allele mining of the *Lhcb1*.

Geographic region	Number of accessions	Countries	Number of countries
Africa	55	Algeria, Eritrea, Egypt, Ethiopia, Libya, Morocco, Tunisia	7
North East Asia	110	Afghanistan, Azerbaijan, China, Cyprus, Georgia, India, Iran, Pakistan, Tajikistan, Turkey, Turkmenistan, Uzbekistan	12
Middle East Asia	56	Iraq, Jordan, Lebanon, Palestine, Syria	5
Arabian Peninsula	14	Oman, Saudi Arabia, Yemen	3
Europe	9	Albania, Bosnia and Herzegovina, Deutschland, France, Greece, Russia, Serbia and Montenego	7
Australia	2	Australia	1
Unkown	46	The country of origin was not known	—
Total	292		35

Genomic DNA of barley accessions was extracted from 200 mg young leaf tissue using a modified CTAB method [24]. DNA from all samples was quantified using a spectrophotometer and normalized to a concentration of 20 ng/µl.

### Evaluation of agronomic traits

All accessions were evaluated for six agronomic traits, flag leaf area (FLA in cm^2^), spike length (SL in cm), number of grains per spike (NGS), leaf color (SPAD value), plant height (PH in cm) and 1000-kernel weight (TKW in g) in field at the Experimental Station of Guangzhou University Guangzhou, Guangdong Province, China (23°16′N; 113°23′E, elevation 16 m asl). The experiments were repeated twice (2009/2010 and 2010/2011) with three replications. Eleven plants per genotype were planted in a single-row plot at 1.5 m long and 30 cm apart. Three randomly selected plants per genotype from each replication were characterized for six traits (Table 2) as described by Gupta [25] and Lakew [26].

**Table 2:** Means ± standard deviations and range of values for six agronomic traits for 292 barley accessions in two growing seasons (2009/2010 and 2010/2011).

Trait	Description	Unit/scale	2009/2010*		2010/2011*	
			Range	Mean	Range	Mean
FLA	Flag leaf area	cm^2^	7.2–91.4	42.1±13.7	—	—
LC	Leaf color	SPAD value	26.3–56.1	43.2±5.8	31.4–65.2	47.5±5.1
PH	Plant height	cm	11.0–79.5	51.1±16.9	44.0–115.5	77.3±12.6
SL	Spike length	cm	5.2–12.6	8.3±1.4	4.6–13.2	8.43±1.43
NGS	Number of grains per spike	No. of grains/spike	2.5–54.7	22.8±11.7	2.3–68.0	31.0±14.6
TGW	Thousand grain weight	g/1,000 grains	13.7–66.2	41.98±9.15	21.3–72.	42.5±9.9

* Because 19 and 21 barley accessions did not head in 2009/2010 and 2010/2011 growing seasons, respectively, measurements of three yield related traits—spike length (SL in cm), number of grains per spike (NGS) and 1000-grain weight (TKW in g)—were conducted for only 273 and 271 barley accessions for the two seasons, respectively.

### Primers for *Lhcb1*

**Figure 1:**
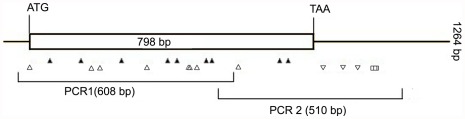
Diagram of PCR amplification and distribution of SNPs in *Lhcb1*. The figure was derived from PARSESNP output files [33]. The relative positions of the two PCR products amplified for EcoTILLING are indicated. White up arrows indicate changes in coding regions of DNA that do not affect the amino acid sequence of the protein product. White down arrows indicate changes to noncoding regions of DNA. Black up arrows indicate changes that induce missense mutations in the predicted protein product. White squares indicate insertions or deletions.

To screen for natural variation in the *Lhcb1* of barley, nested PCR was employed to amplify coding region of the *Lhcb1* as described by Wienholds [27]. The primer design was based on the published mRNA sequence (including complete coding region) of the *Lhcb1* from GeneBank (accession no. AK359563.1) with melting temperatures around 60°C using Primer 5.0 software (Premier Biosoft International, Palo Alto, CA, USA) (Table 3 and Fig. 1). The primer sequences of the gene were attached with an M13F sequence (5′-cacgacgttgtaaaacgac) in 5′-end of forward primers or an M13R sequence (5′- ggataacaatttcacacagg) in 5′-end of reverse primers (Table 3) for second PCR. M13 forward primers labeled with IRDye800 at 5′-end and M13 reverse primers labeled with IRDye700 at 5′-end were synthesized by LI-COR Inc.

**Table 3:** Primer sequences used for PCR amplification of the *Lhcb1* gene.

Primer name	Sequence 5′→3′
Lh608-F	AGGGACAACTCCCGTCTT
Lh608-R	CTGCCTCCAGGATAAAGTG
Lh510-F	GAGATCGTTGACCCACTTTA
Lh510-R	TTAGAGCGCTAGCCTAATTG
M13-Lh608-F	* cacgacgttgtaaaacgac AGGGACAACTCCCGTCTT
M13-Lh608-R	ggataacaatttcacacagg CTGCCTCCAGGATAAAGTG
M13-Lh510-F	cacgacgttgtaaaacgac GAGATCGTTGACCCACTTTA
M13-Lh510-R	ggataacaatttcacacagg TTAGAGCGCTAGCCTAATTG
M13F (IRDye800)	cacgacgttgtaaaacgac
M13R (IRDye700)	ggataacaatttcacacagg

* Primer sequences in lower case are tag sequences (M13F or M13R).

### PCR amplification and EcoTILLING assays

For EcoTILLING assay, the mRNA sequence of the *Lhcb1* was amplified by nested PCR as described by Wienholds [27] with minor modifications. The accession ICARDA IG 26727 was selected as a reference. Initial PCR amplification of the target region was performed using 20 ng of genomic DNA (1∶1 reference to sample DNAs) in a volume of 10 µl containing 1.0 µl of 10×PCR buffer, 0.1 µM of forward and reverse gene-specific primers, 2.5 mM MgCl_2_, 0.4 mM dNTPs, and 0.4 U *Taq* DNA Polymerase (Bio Basic Inc., Toronto, Canada) under the following conditions: 5 min denaturation at 94°C followed by 35 cycles of 30 s at 94°C, 45 s at 58°C and 1 min at 72°C, and a final step of 3 min at 72°C for additional PCR extension. The PCR product was diluted in 90 µl of distilled water as template for second round nested PCR.

The second round of PCR was carried out in a 10 µl solution containing 1 µl of initial PCR product, 1.0 µl 10×PCR buffer, 0.02 µM M13F-tailed gene-specific forward primer, 0.04 µM M13R-tailed gene-specific reverse primer, 0.08 µM IRD800-labeled M13 forward primers, 0.06 µM IRD700-labeled M13 reverse primers, 2.5 mM MgCl_2_, 0.4 µM dNTPs, and 0.04 U *Taq* DNA polymerase (Bio Basic Inc., Toro nto, Canada). Thermocycling conditions consisted of an initial step of 94°C for 1 min followed by 38 cycles of 20 s at 94°C, 30 s at 58°C and 1 min at 72°C, and a final step of 3 min at 72°C. After the nested PCR, heteroduplexes formation was performed by incubating the reaction mix at 99°C for 10 min, followed by 70 cycles starting at 70°C for 20 sec with a decrement of 0.3°C in subsequent cycles and then holding at 4°C.

Heteroduplex DNA was cleaved at 45°C for 15 min in a 20 µl of reaction solution containing 10 µl PCR product, 10 mM HEPES (pH 7.5), 10 mM MgSO_4_, 0.002% (w/v) Triton X-100, 0.2 µg/ml of bovine serum albumin, and 0.4 µl *CEL* I enzyme. *CEL* I enzyme was prepared following Guo and Li [28]. Digestion was stopped by addition of 5 µl of 0.25 M EDTA (pH 8), mixing thoroughly, and then put on ice. Digested products were separated in a LICOR 4300 DNA Analyzer (LICOR, Nebraska, USA) using 6.5% denaturated polyacrylamide gel electrophoresis running at 1500 V, 40 mA, 50 W and 45°C for 5 hours.

**Figure 2:**
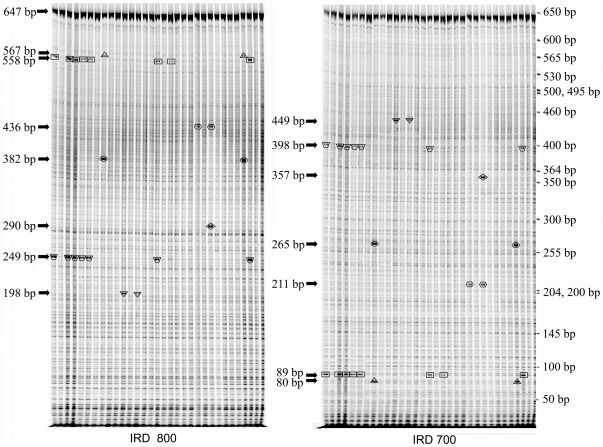
Detection of polymorphisms for part region of the *Lchb1* gene with EcoTILLING. Sampled images of the IRD 700 and IRD 800 channels are shown at right and left, respectively. The specific cleavage products appear as intense dark bands between 80 to 567 bp with molecular weights listed to the left in each channel image by arrows. Complementary fragments in corresponding lanes between the IRD 700 and IRD 800 channel images labeled with the same box pattern (including triangle, rectangle, hexagon, oval, diamond, inverted trapezoid and inverted triangle). The sizes of complementary fragments in the IRD 700 labeled and the IRD 800 labeled add up to the size of PCR fragment (647 bp). Molecular weights are provided by the GelBuddy program. The sizes of DNA ladder are listed to the right of the IRD700 image.

During electrophoresis, the LI-COR DNA analyzer captured two images in IRD700 and IRD800 channels, respectively. Tiff images were manually scored using the GelBuddy program [29]. Big dark bands with different sizes in both IRD700 and IRD800 channels were considered as a polymorphic site (Fig. 2). Total length PCR products from both channels should be equivalent to the fragment size of the undigested PCR product. Data summary reports generated by GelBuddy were imported to Microsoft Excel for further analysis. The number of haplotypes was estimated using Bayesian methods implemented in the program PHASE, version 2.1 [30], [31].

### DNA sequencing and statistical analysis

Once a polymorphism was identified, the corresponding DNA sample was amplified using gene-specific primers. The resulted PCR fragment was directly sequenced. Each polymorphic site was sequenced from more than one accession to confirm that only two alleles segregated at any specific site. Multiple sequence alignment was conducted using ClustalW software (http://www.ebi.ac.uk/tools). The potential effect of SNPs on protein function was predicted using SIFT (Sorting Intolerant from Tolerant) [32] and PARSESNP (Position-Specific Scoring Matrix) programs [33]. Nucleotide diversity (π), haplotype diversity and Tajima's D [34] were calculated using DnaSP v5.0 [35].

### Association between SNPs and agronomic traits

In order to test the effect of SNPs in the *Lhcb1* on agronomic traits of barley, the association between SNP markers and traits was calculated using TASSEL software v3.0 (http://www.maizegenetics.net/tassel). To evaluate population structure, all barley accessions were genotyped with 21 genome-wide SSR molecular markers (3 SSRs for each chromosome) (Table S2), and three groups were defined (unpublished) using Structure software version 2 [36]. These independent group memberships were used as covariates in the genotype–phenotype association analysis with the GLM_Q model. The marker being tested was treated as a fixed effect. The significance of associations between markers and traits was tested using an F-test. The association between a marker and a trait is represented by its R^2^ value, an estimate of the percentage of variance explained by the marker.

## Results

### Allele mining in the *Lhcb1*

EcoTILLING identified 23 natural variation sites in the amplified region of the *Lhcb1* across 292 accessions. The frequency of polymorphic sites ranged from 0.003 to 0.264, with an average of 0.06 per polymorphic site in 292 samples (Table 4). Sequencing random samples containing each of these variation sites confirmed 20 single nucleotide polymorphisms (SNPs) and 3 insert/deletions (indels) in the 23 natural variation sites (Table 4 and Fig. 1). However, variation site was not identified in the two samples that showed two variation sites in EcoTILLING by sequencing (Table 4). The *Lhcb1* has a frequency of one SNP per 49.3 bp in 292 barley accessions. The ratio of transitions (C-T and A-G) to transversions (A-C, A-T, C-G and G-T) of SNPs was 15 to 5 in the targeted region of *Lhcb1*. In 20 sequence validated SNPs, nine sites were missense changes, eight were silent synonymous changes, and three were indels in the 3′ downstream of non-coding region. Two of nine missense changes were predicted to be deleterious to the function of *Lhcb1* protein (Table 4).

**Table 4:** List of nucleotide polymorphisms in *Lhcb1* with their effects on codons, frequencies.

No.	Nucleotide Change^a^	Band^b^	Frequency^c^	Effect^d^	PARSESNP^e^	SIFT^f^
1	G81C	+	0.010	A2 =		
2	G132T	+	0.010	K19N		
3	C220A	+	0.007	P49T		
4	C252T	+	0.031	L59 =		
5	G276A	+	0.233	P67 =		
6	G334A	+	0.007	A87T	8.3	0.16
7	A411G	+	0.051	G112 =		
8	G463A	+	0.058	V130I		
9	T490C	+	0.010	F139L		
10	C531T	+	0.007	L152 =		
11	C534T	+	0.003	V153 =		
12	C550T	+	0.014	L159 =		
13	T572C	+	0.240	V166A	**17.4**	**0.04**
14	G589A	+	0.051	V172I	5.4	0.71
15	C669T	+	0.062	L198 =		
16	G781A	+	0.007	G236S	**22.6**	**0.02**
17	G805A	+	0.062	A244T	5	0.44
18	C907A	+	0.264	Non-coding		
19	T961C	+	0.041	Non-coding		
20	G1006C	+	0.065	Non-coding		
21	*GTGC1049:	+	0.007	Non-coding		
22	*CTGCT1054:	+	0.007	Non-coding		
23	*A1060:	+	0.007	Non-coding		
24	ND	+(∼242 bp)	0.007			
25	ND	+(∼532 bp)	0.007			

a The first letter indicates the common bp at this site, followed by the position of the SNP in the sequence on GenBank accession number AK359563.1, and then the nucleotide which is the rare variant at this site.

b All nucleotide changes identified by sequencing were first by EcoTILLING as a band on the gel image. In two sample, ∼242 bp and ∼532 bp were identified on the EcoTILLING gel for which corresponding polymorphisms could not be confirmed by sequencing.

c Frequency was calculated by dividing the number of similar nucleotide changes identified on the EcoTILLING gel by the number of samples analyzed.

d The first letter indicates the common amino acid at this site, followed by the position of the SNP within the predicted protein sequence and then the amino acid change induced by the variant nucleotide polymorphism. “ = ” means no change in the amino acid encoded by that codon (synonymous variation).

e A non-synonymous SNP is predicted to be damaging to the encoded protein if the PARSESNP score is >10 (bold).

f A non-synonymous SNP is predicted to be damaging to the encoded protein if the SIFT score is <0.05 (bold).

* Adjacent polymorphisms appear as a single band on the gel image.

The nucleotide diversity (π) of the *Lhcb1* was 0.00166 across 292 barley accessions. For different geographic regions, π values ranged from 0.0011 for European accessions (9 accessions) to 0.00212 for Middle East Asian accessions (56 accessions). Similarly, π for SPON was the highest among the three groups, SPON, VUL-LR and VUL-IG (Table 5). Tajima's D statistics was calculated to examine whether the SNPs in the sequenced region of *Lhcb1* were neutrally selected. Resulting Tajima's D value was not significant (*P*<0.05) although a high negative value of −1.12884 was estimated. Thus, the *Lhcb1* in the population did not significantly deviate from neutral selection.

**Table 5:** Barley *Lhcb1* nucleotide diversity (π), haplotype diversity and Tajima's D test for each geographic region and three different genotype groups.

Population		Number of accessions	Number of polymorphic sites	nucleotide diversity (π)	Number of haplotypes	haplotpe diversity	Tajima's D
Total		292	23	0.00166	31	0.819	−1.12884
Geographic region	AFR	55	15	0.00181	12	0.830	−0.91199
	NEA	110	12	0.00122	14	0.734	−0.84835
	MEA	56	16	0.00212	18	0.903	−0.69602
	APS	14	6	0.00171	5	0.791	0.52914
	EUR	9	5	0.00110	3	0.556	−0.103796
	AUS	2	-	-	1	-	-
	UNK	46	12	0.00154	10	0.795	−0.85606
Genotype group	VUL-LR	171	17	0.00155	19	0.804	−0.89987
	VUL-IG	82	12	0.00166	11	0.776	−0.34944
	SPON	39	16	0.00198	18	0.895	−1.09943

AFR: Africa, APS: Arabian Peninsula, AUS: Australia, EUR: Europe, MEA: Middle East Asia, NEA: North East Asia, UNK: the country of origin was not known. SPON: *H. spontaneum*; VUL-LR: *H. vulgare* Landraces, VUL-IG: *H. vulgare* Cultivars or Improved Genotypes.

### Haplotype diversity

For the 23 sequence-validated nucleotide variations including 20 SNPs and 3 indels, 292 accessions demonstrated 31 distinguishable haplotypes (Table S3) with various frequencies among haplotypes (Table 6). The level of haplotype diversity was 0.819. Among the haplotypes, H31, H30, and H29 showed significantly higher frequency than others, with about one-third accessions (93 accessions) carrying H31, one-fourth (68 accessions) carrying H30, and one-seventh carrying (41 accessions) H29. The other 28 haplotypes (from H1 to H28) presented in only one-third of 292 accessions with very low frequencies (0.003∼0.055) for each haplotype (Table 6).

**Table 6:** Frequency of *Lhcb1* haplotypes in different geographic barley growing regions and different genotype groups of barley.

		Geographic regions							Genotype group		
Haplotype	Overall (292)	AFR (55)	NEA (110)	MEA (56)	APS (14)	EUR (9)	AUS (2)	UNK (46)	VUL-LR (171)	VUL-IG (82)	SPON (39)
H1	0.003	-	0.009	-	-	-	-	-	0.006	-	-
H2	0.003	-	-	0.018	-	-	-	-	-	-	0.026
H3	0.003	-	0.009	-	-	-	-	-	-	-	0.026
H4	0.003	0.018	-	-	-	-	-	-	0.006	-	-
H5	0.003	0.018	-	-	-	-	-	-	0.006	-	-
H6	0.003	-	0.009	-	-	-	-	-	0.006	-	-
H7	0.003	-	-	0.018	-	-	-	-	-	-	0.026
H8	0.003	-	-	0.018	-	-	-	-	-	-	0.026
H9	0.003	-	0.009	-	-	-	-	-	-	-	0.026
H10	0.003	-	-	0.018	-	-	-	-	-	-	0.026
H11	0.003	-	-	0.018	-	-	-	-	-	-	0.026
H12	0.003	-	-	-	-	-	-	0.022	-	0.012	-
H13	0.003	0.018	-	-	-	-	-	-	0.006	-	-
H14	0.003	-	0.009	-	-	-	-	-	0.006	-	-
H15	0.007	-	-	-	-	-	-	0.043	-	0.024	-
H16	0.007	-	-	0.036	-	-	-	-	0.012	-	-
H17	0.007	0.018	-	0.018	-	-	-	-	-	-	0.051
H18	0.007	-	-	0.036	-	-	-	-	-	-	0.051
H19	0.010	-	-	0.054	-	-	-	-	-	-	0.077
H20	0.010	-	0.009	0.018	0.071	-	-	-	0.006	-	0.051
H21	0.010	-	0.009	0.018	-	-	-	0.022	0.006	0.012	0.026
H22	0.010	-	0.027	-	-	-	-	-	0.018	-	-
H23	0.017	-	-	0.089	-	-	-	-	0.012	-	0.077
H24	0.021	0.055	-	-	-	-	-	0.065	0.018	0.037	-
H25	0.021	0.055	0.018	0.018	-	-	-	-	0.029	0.012	-
H26	0.031	0.018	-	0.036	**0.357**	-	-	0.022	0.041	0.024	-
H27	0.048	0.018	0.109	-	-	-	-	0.022	0.07	0.012	0.026
H28	0.055	0.073	0.027	0.071	-	0.111	-	0.087	0.012	0.146	0.051
H29	0.140	**0.291**	0.091	0.143	0.071	-	1	0.087	0.187	0.085	0.051
H30	0.233	0.2	0.209	0.179	0.214	**0.667**	-	**0.326**	0.24	0.305	0.051
H31	0.318	0.218	**0.455**	**0.196**	0.286	0.222	-	0.304	**0.316**	**0.329**	**0.308**

Haplotypes are ordered by overall frequency in all barley accessions. AFR: Africa, APS: Arabian Peninsula, AUS: Australia, EUR: Europe, MEA: Middle East Asia, NEA: North East Asia, UNK: the country of origin was not known. SPON: *H. spontaneous*; VUL-LR: *H. vulgare* Landraces, VUL-IG: *H. vulgare* Cultivars or Improved genotypes. The number in bracket indicates the number of plants which were scored and measured. Most frequent haplotypes within each population are highlighted in bold.

The frequencies of the *Lhcb1* haplotypes also differed significantly among the geographical regions of tested accessions (Table 6). This was particularly obvious for haplotype H26, which is most frequent in the Arabian Peninsula (0.357), but rare in African (0.018) and Middle East Asia (0.036) and completely absent in Australia, North East Asia and Europe. These rare haplotypes were usually confined to specific geographic regions. Of the 28 rare haplotypes (<10% in the accessions sampled), 20 were unique to only one region with nine accessions exclusively from Middle East Asia, six from North East Asia, three from Africa, and two without information on their origins. The *Lhcb1* haplotype diversity for each geographic region ranged from 0.556 (Europe) to 0.903 (Middle East Asia) with a mean of 0.768 (Table 5). These values in general corresponded to the number of *Lhcb1* haplotypes discovered with some exceptions. For example, accessions from Middle East Asia had the highest haplotype diversity of *Lhcb1*, and also the most *Lhcb1* haplotypes (n = 18). However, the accessions from North East Asia had a very low haplotype diversity value, but the *Lhcb1* haplotypes (n = 14) second to Middle East Asia due to majority of low-frequency haplotypes in this region.

In addition, significant difference in *Lhcb1* haplotype diversity was observed among three barley groups, i. e. SPON, VUL-LR and VUL-IG (Table 5), with SPON having the highest haplotype diversity (Table 5). Although three groups had six haplotypes in common, SPON, VUL-LR and VUL-IG each had ten, seven and two unique haplotypes, respectively (Table 6).

### Association between SNPs and phenotypic traits

Association analysis was performed to find tentative association between nucleotide variations in *Lhcb1* with agronomic traits. Because 14 SNPs were either linkage disequilibrium (LD) within subgroups or rare alleles (frequency <3%), only nine distinct SNPs were used for association analysis. Among them, five SNPs were significantly associated (*P*<0.01) with one or two phenotypic traits, with one SNP that were highly significantly associated (*P*<0.001) with two phenotypic traits (Table 7). The percentage of variation of a given trait explained by each associated SNP was up to 8.0% with an average of 3.9%. The SNP at position 907 bp in the *Lhcb1* was highly associated with SL and NGS (*P*<0.001), and explained 8.0% or 5.3% and 5.0% or 5.6% of the variation for SL and NGS in both seasons, respectively. Another SNP at position 1006 bp exhibited significant association (*P*<0.01) with SL, explaining 2.7% and 2.6% phenotypic variation for the SL in both seasons. The SNP at position 463 bp was significantly associated (*P*<0.01) with FLA and LC, explaining 3.0% and 2.2% phenotypic variation for the FLA in season one and LC in season two. Two SNPs (positions 589 bp and 961 bp) were significantly associated (*P*<0.01) with TGW, both explaining approximately 2.4% phenotypic variation in 2009 and 2010 experiments.

**Table 7:** Significant association between SNPs of *Lhcb1* and agronomic traits of barley.

Growing seasons	Traits	SNPs position	F	P	R^2^	Excellent allele	Frequency of excellent allele
2009/2010	SL	907C>A	24.85**	0.000001	0.080	A	24.18%
	NGS	907C>A	15.54**	0.000103	0.050	C	75.82%
	FLA	463G>A	10.25*	0.001522	0.030	A	5.82%
	SL	1006G>C	7.92*	0.005244	0.027	G	93.04%
	TGW	961T>C	7.60*	0.006230	0.024	C	4.01%
	TGW	589G>A	7.52*	0.006511	0.024	A	5.49%
2010/2011	NGS	907C>A	16.76**	0.0000563	0.056	C	75.28%
	SL	907C>A	15.62**	0.0000996	0.053	A	24.72%
	LC	463G>A	7.46*	0.006699	0.022	G	94.18%
	SL	1006G>C	7.29*	0.007374	0.026	G	92.99%

FLA, flag leaf area (cm^2^); NGS, number of grains per spike; LC, leaf color (SPAD); PH, plant height (cm); SL, spike length (cm); TKW, Thousand grain weight (g).

The number of SNP positions is relative to the sequence on GenBank accession number AK359563.1.

R^2^ is the fraction of the total variation explained by the marker.

* (P<0.01) indicates the SNP significantly associated with traits.

** (P<0.001) indicates the SNP highly significantly associated with traits.

## Discussion

### Use of EcoTILLING to discover SNP for specific genes in barley

EcoTILLING was initially used to characterize the variability of genes within a collection of Arabidopsis ecotypes [17]. Since then, it has been successfully used in the analysis of natural variability of in *Populus trichocarpa* [37], in wheat [19], in *Brassica* [20] and in barley [22], [23]. Used in combination with sequencing, EcoTILLING becomes a fast, reliable, economical method for identifying polymorphisms and developing functional markers for plants [38]. Once polymorphisms are identified by EcoTILLING, individuals can be grouped according to haplotype and only interesting haplotypes and/or representatives from each haplotype need to be sequenced. In addition, EcoTILLING points at the approximate location of the polymorphism within the locus studied and, therefore, restricts the necessity of sequencing the complete locus but only the regions around the polymorphic sites [39]. In this study, all these advantages account for a reduction of more than 85% in number of sequencing reactions potentially required to identify the variability of the *Lhcb1* in the germplasm collection.

### Nucleotide of variation in *Lhcb1*

Many *LHCP* from various plant species have been identified by transcriptome analysis. However, the allelic variation in *LHCP* has not been systematically characterized. Fusari [21] found seven SNPs and two indels in a sunflower *LHCP* after screening 19 elite inbred lines using EcoTILLING. Our primary goal was to characterize genetic variation of an *Lhcb1* in barley. To this end, a set of barley accessions originated from several geographic regions was selected for allele mining. EcoTILLING revealed 23 nucleotide changes including 20 SNPs and 3 indels in the *Lhcb1*, which formed 31 haplotypes in 292 accessions. Compared to previous report on an *Lhcb2* in 24 unrelated black poplar [40], the nucleotide diversity (π = 0.00166) and haplotype diversity (0.819) of the *Lhcb1* was lower. The average frequency of SNPs was 1 per 49.3 bp, which was higher than reported on an *Lhcb2* (1SNP/73.9 bp) [40] and on a *LHCP* (1SNP/76.7 bp) [21]. In addition, Middle East Asia was identified as a hotspot of the haplotype diversity (0.903) (Table 5), which is in agreement with several earlier reports that the barley accessions [41], [42] and wheat accessions [43] from Middle East Asia had high genetic diversity. Among the three gene pools, SPON, VUL-LR and VUL-IG, SPON showed the highest nucleotide diversity (π) and the highest haplotype diversity in the *Lhcb1* in this study, which supports the earlier observations of high genetic diversity in SPON [44]–[46].

### Association between SNPs of *Lhcb1* and agronomic traits

*LHCP* family in plants encodes many LHCPs that play essential roles in light capture and photoprotection in the photosystem. A strong relationship between the photosynthetic capacity and grain yield was observed in cereals such as wheat and maize [47], [48]. It is critical that the photosynthetic capacities of both the total canopy and specific leaves are maintained throughout the entire plant life cycle, especially from flowering to grain maturity [49]. In agronomic terms, some ‘stay green’ mutants have higher kernel weights than wide type in maize. Thus ‘stay green’ traits have extensively used in improving grain yield under stress conditions such as drought and heat. However, little is known about the underlying genetics and molecular biology of the trait(s) even though some analyses have been performed in maize and sorghum [49], [50].

Association analysis emerged as a powerful approach to search for the role of genetic polymorphisms in phenotype variations in responses to environmental stresses [51]–[53]. In this study, five SNPs in barley *Lhcb1* were significantly associated with at least one agronomic trait. Of these five SNPs, two at positions 463 bp and 589 bp of *Lhcb1* were missense mutations, but they did not severely affect protein function according to SIFT, and other three SNPs at positions 907 bp, 961 bp and 1006 bp were in a non-coding region. Due to low minor allele frequency, association data for three of these five SNPs at positions 463 bp, 589 bp and 961 bp should be interpreted with caution and need to be validated for individual cultivars involved in crosses before they can be applied to marker-assisted selection [54], [55]. Further research on relationship between these newly detected SNPs in the *Lhcb1* and other important agronomic traits may provide useful markers as selection tools to improve barley yield under stress conditions.

In conclusion, we have demonstrated EcoTILLING as an efficient approach for allele mining of barley candidate genes. Haplotype sequencing confirmed 23 nucleotide mutantions including 20 SNPs and 3 indels with 31 unique haplotypes in the *Lhcb1* among 292 barley accessions from 35 countries. The results indicated that the accessions from Middle East Asia had the highest nucleotide diversity in the *Lhcb1*, and *H. spontaneum* exhibited greater genetic diversity than *H. vulgare*. Thus introgression of genes from Middle East Asian accessions or *H. spontaneum* in to cultivated barley may enhance genetic diversity. Association analysis showed that five SNPs in the *Lhcb1* were significantly associated with at least one agronomic trait and these SNPs can be used in future studies to assess their usefulness as selection criteria for improving these agronomic traits.

## Supporting Information

Table S1 **General information of barley accessions used in this study.**
(DOC)

Table S2 **The information of SSR markers used in evaluation of population structure.**
(DOC)

Table S3 **Distribution of polymorphic SNPs across hyplotypes.** SNPs relative to the most common sequence (haplotype H31) are indicated in boldface. The number of SNP positions is relative to the sequence on GenBank accession number AK359563.1. A *horizontal dash* indicates the absence of the indicated bases.
(DOC)
