# Supplementary material for: Allelic Variations of a Light Harvesting Chlorophyll A/B-Binding Protein Gene (Lhcb1) Associated with Agronomic Traits in Barley
Source: PLoS One. 2012 May 25;7(5):e37573. doi: 10.1371/journal.pone.0037573 (PMC3360778; doi:10.1371/journal.pone.0037573)
Supplement: Table S1 — General information of barley accessions used in this study. (DOC) [file pone.0037573.s001.doc]

**Table S1** General information of barley accessions used in this study

| ICARDA IG | Origin country^a^ | Donor county | Genotype description (species) | Ear type |
| --- | --- | --- | --- | --- |
| 16981 | TKM | USA | *Hordeum vulgare* subsp. vulgare convar. vulgare | 6-row |
| 17424 | SCG | USA | *Hordeum vulgare* subsp. vulgare convar. vulgare | 6-row |
| 18767 | TUR | USA | *Hordeum vulgare* subsp. vulgare convar. vulgare | 6-row |
| 18983 | GRC | USA | *Hordeum vulgare* subsp. vulgare convar. vulgare | 6-row |
| 19390 | CHN | USA | *Hordeum vulgare* subsp. vulgare convar. vulgare | 6-row |
| 19391 | CHN | USA | *Hordeum vulgare* subsp. vulgare convar. vulgare | 6-row |
| 19453 | IRN | USA | *Hordeum vulgare* subsp. vulgare convar. vulgare | 6-row |
| 19687 | CHN | USA | *Hordeum vulgare* subsp. vulgare convar. vulgare | 6-row |
| 20905 | AFG | USA | *Hordeum vulgare* subsp. vulgare convar. vulgare | 6-row |
| 23248 | ETH | USA | *Hordeum vulgare* subsp. vulgare convar. vulgare | 6-row |
| 23515 | ETH | USA | *Hordeum vulgare* subsp. vulgare convar. vulgare | 6-row |
| 24634 | DZA | USA | *Hordeum vulgare* subsp. vulgare convar. vulgare | 6-row |
| 24745 | AZE | USA | *Hordeum vulgare* subsp. vulgare convar. vulgare | 6-row |
| 24746 | TKM | USA | *Hordeum vulgare* subsp. vulgare convar. vulgare | 6-row |
| 24751 | CHN | USA | *Hordeum vulgare* subsp. vulgare convar. vulgare | 6-row |
| 24764 | CHN | USA | *Hordeum vulgare* subsp. vulgare convar. vulgare | 6-row |
| 24774 | CHN | USA | *Hordeum vulgare* subsp. vulgare convar. vulgare | 6-row |
| 24944 | CHN | USA | *Hordeum vulgare* subsp. vulgare convar. vulgare | 6-row |
| 24953 | LBY | USA | *Hordeum vulgare* subsp. vulgare convar. vulgare | 6-row |
| 25704 | IND | USA | *Hordeum vulgare* subsp. vulgare convar. vulgare | 6-row |
| 25839 | EGY | USA | *Hordeum vulgare* subsp. vulgare convar. vulgare | 6-row |
| 25883 | AFG | USA | *Hordeum vulgare* subsp. vulgare convar. vulgare | 6-row |
| 26002 | AZE | USA | *Hordeum vulgare* subsp. vulgare convar. vulgare | 6-row |
| 26055 | CHN | USA | *Hordeum vulgare* subsp. vulgare convar. vulgare | 6-row |
| 26056 | CHN | USA | *Hordeum vulgare* subsp. vulgare convar. vulgare | 6-row |
| 26081 | CHN | USA | *Hordeum vulgare* subsp. vulgare convar. vulgare | 6-row |
| 26172 | CHN | USA | *Hordeum vulgare* subsp. vulgare convar. vulgare | 6-row |
| 26178 | CHN | USA | *Hordeum vulgare* subsp. vulgare convar. vulgare | 6-row |
| 26229 | CHN | USA | *Hordeum vulgare* subsp. vulgare convar. vulgare | 6-row |
| 26276 | IRQ | USA | *Hordeum vulgare* subsp. vulgare convar. vulgare | 6-row |
| 26727 | AZE | USA | *Hordeum vulgare* subsp. vulgare convar. vulgare | 6-row |
| 27076 | CHN | USA | *Hordeum vulgare* subsp. vulgare convar. vulgare | 6-row |
| 27630 | PAK | USA | *Hordeum vulgare* subsp. vulgare convar. vulgare | 6-row |
| 27649 | IRN | USA | *Hordeum vulgare* subsp. vulgare convar. vulgare | 6-row |
| 27653 | IRN | USA | *Hordeum vulgare* subsp. vulgare convar. vulgare | 6-row |
| 27683 | AFG | USA | *Hordeum vulgare* subsp. vulgare convar. vulgare | 6-row |
| 27773 | IRN | USA | *Hordeum vulgare* subsp. vulgare convar. vulgare | 6-row |
| 27790 | IRN | USA | *Hordeum vulgare* subsp. vulgare convar. vulgare | 6-row |
| 27794 | IRN | USA | *Hordeum vulgare* subsp. vulgare convar. vulgare | 6-row |
| 27799 | AFG | USA | *Hordeum vulgare* subsp. vulgare convar. vulgare | 6-row |
| 27803 | AFG | USA | *Hordeum vulgare* subsp. vulgare convar. vulgare | 6-row |
| 29097 | JOR | SYR | *Hordeum vulgare* subsp. vulgare convar. vulgare | 6-row |
| 31406 | SYR | SYR | *Hordeum vulgare* subsp. vulgare convar. vulgare | 6-row |
| 31410 | SYR | SYR | *Hordeum vulgare* subsp. vulgare convar. vulgare | 6-row |
| 31870 | MAR | MAR | *Hordeum vulgare* subsp. vulgare convar. vulgare | 6-row |
| 31876 | MAR | MAR | *Hordeum vulgare* subsp. vulgare convar. vulgare | 6-row |
| 31923 | MAR | MAR | *Hordeum vulgare* subsp. vulgare convar. vulgare | 6-row |
| 31938 | MAR | MAR | *Hordeum vulgare* subsp. vulgare convar. vulgare | 6-row |
| 32475 | EGY | SYR | *Hordeum vulgare* subsp. vulgare convar. vulgare | 6-row |
| 32482 | EGY | SYR | *Hordeum vulgare* subsp. vulgare convar. vulgare | 6-row |
| 32488 | EGY | SYR | *Hordeum vulgare* subsp. vulgare convar. vulgare | 6-row |
| 32601 | PAK | SYR | *Hordeum vulgare* subsp. vulgare convar. vulgare | 6-row |
| 32608 | PAK | SYR | *Hordeum vulgare* subsp. vulgare convar. vulgare | 6-row |
| 32618 | PAK | SYR | *Hordeum vulgare* subsp. vulgare convar. vulgare | 6-row |
| 32708 | SYR | SYR | *Hordeum vulgare* subsp. vulgare convar. vulgare | 6-row |
| 32711 | SYR | SYR | *Hordeum vulgare* subsp. vulgare convar. vulgare | 6-row |
| 32826 | OMN | ITA | *Hordeum vulgare* subsp. vulgare convar. vulgare | 6-row |
| 32954 | OMN | GBR | *Hordeum vulgare* subsp. vulgare convar. vulgare | 6-row |
| 32962 | OMN | ITA | *Hordeum vulgare* subsp. vulgare convar. vulgare | 6-row |
| 32971 | OMN | ITA | *Hordeum vulgare* subsp. vulgare convar. vulgare | 6-row |
| 32977 | OMN | ITA | *Hordeum vulgare* subsp. vulgare convar. vulgare | 6-row |
| 33024 | DZA | SYR | *Hordeum vulgare* subsp. vulgare convar. vulgare | 6-row |
| 33055 | DZA | SYR | *Hordeum vulgare* subsp. vulgare convar. vulgare | 6-row |
| 33102 | DZA | SYR | *Hordeum vulgare* subsp. vulgare convar. vulgare | 6-row |
| 33195 | CHN | CHN | *Hordeum vulgare* subsp. vulgare convar. vulgare | 6-row |
| 33608 | CHN | CHN | *Hordeum vulgare* subsp. vulgare convar. vulgare | 6-row |
| 33649 | CHN | CHN | *Hordeum vulgare* subsp. vulgare convar. vulgare | 6-row |
| 34263 | CHN | CHN | *Hordeum vulgare* subsp. vulgare convar. vulgare | 6-row |
| 35382 | DZA | SYR | *Hordeum vulgare* subsp. vulgare convar. vulgare | 6-row |
| 35385 | DZA | SYR | *Hordeum vulgare* subsp. vulgare convar. vulgare | 6-row |
| 35386 | DZA | SYR | *Hordeum vulgare* subsp. vulgare convar. vulgare | 6-row |
| 35792 | CHN | CAN | *Hordeum vulgare* subsp. vulgare convar. vulgare | 6-row |
| 35794 | CHN | CAN | *Hordeum vulgare* subsp. vulgare convar. vulgare | 6-row |
| 35800 | CHN | CAN | *Hordeum vulgare* subsp. vulgare convar. vulgare | 6-row |
| 35803 | CHN | CAN | *Hordeum vulgare* subsp. vulgare convar. vulgare | 6-row |
| 35806 | CHN | CAN | *Hordeum vulgare* subsp. vulgare convar. vulgare | 6-row |
| 35808 | CHN | CAN | *Hordeum vulgare* subsp. vulgare convar. vulgare | 6-row |
| 35814 | CHN | CAN | *Hordeum vulgare* subsp. vulgare convar. vulgare | 6-row |
| 35820 | CHN | CAN | *Hordeum vulgare* subsp. vulgare convar. vulgare | 6-row |
| 35822 | CHN | CAN | *Hordeum vulgare* subsp. vulgare convar. vulgare | 6-row |
| 35823 | CHN | CAN | *Hordeum vulgare* subsp. vulgare convar. vulgare | 2-row |
| 35826 | CHN | CAN | *Hordeum vulgare* subsp. vulgare convar. vulgare | 6-row |
| 36052 | LBY | SYR | *Hordeum vulgare* subsp. vulgare convar. vulgare | 6-row |
| 37525 | PAK | USA | *Hordeum vulgare* subsp. vulgare convar. vulgare | 6-row |
| 37554 | LBY | ITA | *Hordeum vulgare* subsp. vulgare convar. vulgare | 6-row |
| 37556 | LBY | ITA | *Hordeum vulgare* subsp. vulgare convar. vulgare | 6-row |
| 37576 | LBY | ITA | *Hordeum vulgare* subsp. vulgare convar. vulgare | 6-row |
| 37726 | TUN | TUN | *Hordeum vulgare* subsp. vulgare convar. vulgare | 6-row |
| 37729 | TUN | TUN | *Hordeum vulgare* subsp. vulgare convar. vulgare | 6-row |
| 37784 | TUN | TUN | *Hordeum vulgare* subsp. vulgare convar. vulgare | 6-row |
| 37813 | TUN | TUN | *Hordeum vulgare* subsp. vulgare convar. vulgare | 6-row |
| 38214 | CHN | CAN | *Hordeum vulgare* subsp. vulgare convar. vulgare | 6-row |
| 107010 | IRN | SYR | *Hordeum vulgare* subsp. vulgare convar. vulgare | 6-row |
| 107020 | IRN | SYR | *Hordeum vulgare* subsp. vulgare convar. vulgare | 6-row |
| 108499 | PAK | SYR | *Hordeum vulgare* subsp. vulgare convar. vulgare | 6-row |
| 108911 | IRQ | IRQ | *Hordeum vulgare* subsp. vulgare convar. vulgare | 6-row |
| 112483 | GEO | DEU | *Hordeum vulgare* subsp. vulgare convar. vulgare | 6-row |
| 112715 | IRN | DEU | *Hordeum vulgare* subsp. vulgare convar. vulgare | 6-row |
| 112840 | LBY | DEU | *Hordeum vulgare* subsp. vulgare convar. vulgare | 6-row |
| 112865 | LBY | DEU | *Hordeum vulgare* subsp. vulgare convar. vulgare | 6-row |
| 112931 | TUR | DEU | *Hordeum vulgare* subsp. vulgare convar. vulgare | 6-row |
| 113084 | SAU | ITA | *Hordeum vulgare* subsp. vulgare convar. vulgare | 2-row |
| 113120 | IRN | IRN | *Hordeum vulgare* subsp. vulgare convar. vulgare | 6-row |
| 113126 | IRN | IRN | *Hordeum vulgare* subsp. vulgare convar. vulgare | 6-row |
| 113128 | IRN | IRN | *Hordeum vulgare* subsp. vulgare convar. vulgare | 6-row |
| 115919 | LBY | DEU | *Hordeum vulgare* subsp. vulgare convar. vulgare | 6-row |
| 120565 | TKM | RUS | *Hordeum vulgare* subsp. vulgare convar. vulgare | 6-row |
| 123901 | UZB | SYR | *Hordeum vulgare* subsp. vulgare convar. vulgare | 6-row |
| 123923 | UZB | SYR | *Hordeum vulgare* subsp. vulgare convar. vulgare | 6-row |
| 125827 | AZE | RUS | *Hordeum vulgare* subsp. vulgare convar. vulgare | 6-row |
| 128122 | IRN | SYR | *Hordeum vulgare* subsp. vulgare convar. vulgare | 6-row |
| 128124 | IRN | SYR | *Hordeum vulgare* subsp. vulgare convar. vulgare | 6-row |
| 128125 | IRN | SYR | *Hordeum vulgare* subsp. vulgare convar. vulgare | 6-row |
| 128133 | IRN | SYR | *Hordeum vulgare* subsp. vulgare convar. vulgare | 6-row |
| 128158 | PAK | SYR | *Hordeum vulgare* subsp. vulgare convar. vulgare | 6-row |
| 128159 | PAK | SYR | *Hordeum vulgare* subsp. vulgare convar. vulgare | 6-row |
| 128160 | PAK | SYR | *Hordeum vulgare* subsp. vulgare convar. vulgare | 6-row |
| 128170 | DZA | SYR | *Hordeum vulgare* subsp. vulgare convar. vulgare | 6-row |
| 128187 | EGY | SYR | *Hordeum vulgare* subsp. vulgare convar. vulgare | 6-row |
| 128199 | JOR | SYR | *Hordeum vulgare* subsp. vulgare convar. vulgare | 6-row |
| 128204 | EGY | SYR | *Hordeum vulgare* subsp. vulgare convar. vulgare | 6-row |
| 128218 | LBY | SYR | *Hordeum vulgare* subsp. vulgare convar. vulgare | 6-row |
| 135258 | JOR | SYR | *Hordeum vulgare* subsp. vulgare convar. vulgare | 6-row |
| 135528 | TKM | SYR | *Hordeum vulgare* subsp. vulgare convar. vulgare | 6-row |
| 137761 | TJK | SYR | *Hordeum vulgare* subsp. vulgare convar. vulgare | 6-row |
| 22957 | ETH | USA | *Hordeum vulgare* subsp. vulgare convar. distichon | 2-row |
| 27892 | SAU | USA | *Hordeum vulgare* subsp. vulgare convar. distichon | 2-row |
| 17406 | BIH | USA | *Hordeum vulgare* subsp. vulgare convar. vulgare | 2-row |
| 17410 | BIH | USA | *Hordeum vulgare* subsp. vulgare convar. vulgare | 2-row |
| 19620 | ALB | USA | *Hordeum vulgare* subsp. vulgare convar. vulgare | 2-row |
| 20900 | AFG | USA | *Hordeum vulgare* subsp. vulgare convar. vulgare | 2-row |
| 22912 | ETH | USA | *Hordeum vulgare* subsp. vulgare convar. vulgare | 2-row |
| 24720 | TKM | USA | *Hordeum vulgare* subsp. vulgare convar. vulgare | 2-row |
| 25095 | CHN | USA | *Hordeum vulgare* subsp. vulgare convar. vulgare | 2-row |
| 25327 | CHN | USA | *Hordeum vulgare* subsp. vulgare convar. vulgare | 2-row |
| 25710 | IND | USA | *Hordeum vulgare* subsp. vulgare convar. vulgare | 2-row |
| 25843 | EGY | USA | *Hordeum vulgare* subsp. vulgare convar. vulgare | 2-row |
| 25947 | CHN | USA | *Hordeum vulgare* subsp. vulgare convar. vulgare | 2-row |
| 25961 | CHN | USA | *Hordeum vulgare* subsp. vulgare convar. vulgare | 2-row |
| 27655 | IRN | USA | *Hordeum vulgare* subsp. vulgare convar. vulgare | 2-row |
| 27784 | IRN | USA | *Hordeum vulgare* subsp. vulgare convar. vulgare | 2-row |
| 28674 | TUR | USA | *Hordeum vulgare* subsp. vulgare convar. vulgare | 2-row |
| 28677 | TUR | USA | *Hordeum vulgare* subsp. vulgare convar. vulgare | 2-row |
| 28693 | TUR | USA | *Hordeum vulgare* subsp. vulgare convar. vulgare | 2-row |
| 28865 | DEU | USA | *Hordeum vulgare* subsp. vulgare convar. vulgare | 2-row |
| 29057 | SYR | SYR | *Hordeum vulgare* subsp. vulgare convar. vulgare | 2-row |
| 31396 | SYR | SYR | *Hordeum vulgare* subsp. vulgare convar. vulgare | 2-row |
| 31412 | SYR | SYR | *Hordeum vulgare* subsp. vulgare convar. vulgare | 2-row |
| 32687 | EGY | SYR | *Hordeum vulgare* subsp. vulgare convar. vulgare | 2-row |
| 32694 | EGY | SYR | *Hordeum vulgare* subsp. vulgare convar. vulgare | 2-row |
| 32756 | SYR | SYR | *Hordeum vulgare* subsp. vulgare convar. vulgare | 2-row |
| 32774 | SYR | SYR | *Hordeum vulgare* subsp. vulgare convar. vulgare | 2-row |
| 32812 | EGY | SYR | *Hordeum vulgare* subsp. vulgare convar. vulgare | 2-row |
| 32814 | OMN | ITA | *Hordeum vulgare* subsp. vulgare convar. vulgare | 2-row |
| 32978 | OMN | ITA | *Hordeum vulgare* subsp. vulgare convar. vulgare | 2-row |
| 33094 | SYR | SYR | *Hordeum vulgare* subsp. vulgare convar. vulgare | 2-row |
| 35220 | SYR | SYR | *Hordeum vulgare* subsp. vulgare convar. vulgare | 2-row |
| 35236 | SYR | SYR | *Hordeum vulgare* subsp. vulgare convar. vulgare | 2-row |
| 36058 | TJK | SYR | *Hordeum vulgare* subsp. vulgare convar. vulgare | 2-row |
| 37608 | YEM | ITA | *Hordeum vulgare* subsp. vulgare convar. vulgare | 2-row |
| 37612 | YEM | ITA | *Hordeum vulgare* subsp. vulgare convar. vulgare | 2-row |
| 112781 | IRN | DEU | *Hordeum vulgare* subsp. vulgare convar. vulgare | 2-row |
| 113076 | SAU | ITA | *Hordeum vulgare* subsp. vulgare convar. vulgare | 2-row |
| 113082 | SAU | ITA | *Hordeum vulgare* subsp. vulgare convar. vulgare | 2-row |
| 113095 | YEM | ITA | *Hordeum vulgare* subsp. vulgare convar. vulgare | 2-row |
| 128088 | AFG | SYR | *Hordeum vulgare* subsp. vulgare convar. vulgare | 2-row |
| 128172 | SYR | SYR | *Hordeum vulgare* subsp. vulgare convar. vulgare | 2-row |
| 128173 | SYR | SYR | *Hordeum vulgare* subsp. vulgare convar. vulgare | 2-row |
| 128200 | JOR | SYR | *Hordeum vulgare* subsp. vulgare convar. vulgare | 2-row |
| 128202 | JOR | SYR | *Hordeum vulgare* subsp. vulgare convar. vulgare | 2-row |
| 131668 | TJK | SYR | *Hordeum vulgare* subsp. vulgare convar. vulgare | 2-row |
| 38215 | CHN | CAN | *Hordeum vulgare* subsp. spontaneum | 2-row |
| 38611 | SYR | SYR | *Hordeum vulgare* subsp. spontaneum | 2-row |
| 38638 | SYR | ITA | *Hordeum vulgare* subsp. spontaneum | 2-row |
| 38660 | AFG | USA | *Hordeum vulgare* subsp. spontaneum | 2-row |
| 38669 | AFG | USA | *Hordeum vulgare* subsp. spontaneum | 2-row |
| 38672 | TUR | USA | *Hordeum vulgare* subsp. spontaneum | 2-row |
| 38693 | PAK | SYR | *Hordeum vulgare* subsp. spontaneum | 2-row |
| 38956 | PAL | USA | *Hordeum vulgare* subsp. spontaneum | 2-row |
| 39126 | PAL | USA | *Hordeum vulgare* subsp. spontaneum | 2-row |
| 39540 | LBN | USA | *Hordeum vulgare* subsp. spontaneum | 2-row |
| 39802 | PAK | SWE | *Hordeum vulgare* subsp. spontaneum | 2-row |
| 39847 | SYR | SYR | *Hordeum vulgare* subsp. spontaneum | 2-row |
| 39857 | SYR | SYR | *Hordeum vulgare* subsp. spontaneum | 2-row |
| 39891 | EGY | SYR | *Hordeum vulgare* subsp. spontaneum | 2-row |
| 40019 | JOR | SYR | *Hordeum vulgare* subsp. spontaneum | 2-row |
| 40021 | JOR | SYR | *Hordeum vulgare* subsp. spontaneum | 2-row |
| 40022 | JOR | SYR | *Hordeum vulgare* subsp. spontaneum | 2-row |
| 40031 | JOR | SYR | *Hordeum vulgare* subsp. spontaneum | 2-row |
| 40034 | JOR | SYR | *Hordeum vulgare* subsp. spontaneum | 2-row |
| 40035 | JOR | SYR | *Hordeum vulgare* subsp. spontaneum | 2-row |
| 40039 | JOR | SYR | *Hordeum vulgare* subsp. spontaneum | 2-row |
| 40056 | JOR | SYR | *Hordeum vulgare* subsp. spontaneum | 2-row |
| 40059 | JOR | SYR | *Hordeum vulgare* subsp. spontaneum | 2-row |
| 40064 | JOR | SYR | *Hordeum vulgare* subsp. spontaneum | 2-row |
| 40071 | JOR | SYR | *Hordeum vulgare* subsp. spontaneum | 2-row |
| 40072 | JOR | SYR | *Hordeum vulgare* subsp. spontaneum | 2-row |
| 40082 | SYR | SYR | *Hordeum vulgare* subsp. spontaneum | 2-row |
| 40101 | TKM | SYR | *Hordeum vulgare* subsp. spontaneum | 2-row |
| 40104 | TKM | SYR | *Hordeum vulgare* subsp. spontaneum | 2-row |
| 107046 | IRN | SYR | *Hordeum vulgare* subsp. spontaneum | 2-row |
| 107427 | IRQ | SYR | *Hordeum vulgare* subsp. spontaneum | 2-row |
| 110742 | SYR | SYR | *Hordeum vulgare* subsp. spontaneum | 2-row |
| 112787 | IRN | DEU | *Hordeum vulgare* subsp. spontaneum | 2-row |
| 115781 | JOR | SYR | *Hordeum vulgare* subsp. spontaneum | 2-row |
| 120794 | TKM | JPN | *Hordeum vulgare* subsp. spontaneum | 2-row |
| 132606 | AZE | SYR | *Hordeum vulgare* subsp. spontaneum | 2-row |
| 135507 | TKM | SYR | *Hordeum vulgare* subsp. spontaneum | 2-row |
| 135536 | TKM | SYR | *Hordeum vulgare* subsp. spontaneum | 2-row |
| 135624 | TKM | SYR | *Hordeum vulgare* subsp. spontaneum | 2-row |
| 138223 | UNK | SYR | Zanbakian | 2-row |
| 138218 | UNK | SYR | WI 2291 | 2-row |
| 138272 | UNK | SYR | UM (Zanbaka//SLB45-40/H.spont.41-1) | 2-row |
| 138274 | UNK | SYR | UM (SLB39-39/H.spont.41-5) | 2-row |
| 138273 | UNK | SYR | UM (SLB12-59//SLB45-40/H.spont.41-5) | 2-row |
| 138277 | UNK | SYR | UM (SLB05-96/H.spont.41-5) | 2-row |
| 138276 | UNK | SYR | UM (SLB05-96//H.spont.41-1/Tadmor) | 2-row |
| 138270 | UNK | SYR | UM (PI386540/ArabiAbiad//H.spont.41-1/Tadmor) | 2-row |
| 138255 | LBY | SYR | UM (M126/CM67//As/Pro/3/Alanda) | 6-row |
| 138271 | UNK | SYR | UM (Hml//H.spont.41-1/Tadmor) | 2-row |
| 138214 | UNK | SYR | UM (Harmal-02//Esp/1808-4L (P2)) | 2-row |
| 138275 | UNK | SYR | UM (H.spont.41-5/Tadmor//Hml-02/Lignee131) | 2-row |
| 138268 | UNK | SYR | UM (H.spont.41-1/Tadmor) | 2-row |
| 138266 | UNK | SYR | UM (Arta//H.spont.41-5/Tadmor) | 2-row |
| 138267 | UNK | SYR | UM (Arta//H.spont.41-5/Tadmor) | 2-row |
| 138211 | UNK | SYR | UM | 2-row |
| 138212 | UNK | SYR | UM | 2-row |
| 138213 | UNK | SYR | UM | 2-row |
| 138269 | UNK | SYR | UM | 2-row |
| 138220 | UNK | SYR | SLB 05-96 | 2-row |
| 138242 | UNK | SYR | Salmas | 2-row |
| 138251 | UNK | SYR | Sadik-2 | 2-row |
| 138250 | UNK | SYR | Sadik-1 | 2-row |
| 138252 | UNK | SYR | Pamir 9 | 2-row |
| 138221 | UNK | SYR | Moroc 9-75 | 2-row |
| 138240 | UNK | SYR | Matnan-01 | 6-row |
| 138224 | UNK | SYR | Harmal | 2-row |
| 138217 | UNK | SYR | ER/Apm | 2-row |
| 138237 | TUR | SYR | CV (Tokak) | 2-row |
| 138230 | DZA | SYR | CV (Tichedrett) | 6-row |
| 138265 | LBY | SYR | CV (Tarida) | 2-row |
| 138262 | ETH | SYR | CV (Shege) | 6-row |
| 138229 | DZA | SYR | CV (Saida) | 6-row |
| 138236 | JOR | SYR | CV (Rum) | 6-row |
| 138238 | RUS | SYR | CV (Radical) | 2-row |
| 138228 | TUN | SYR | CV (Martin) | 6-row |
| 138244 | CYP | SYR | CV (Mari/Aths*2) | 6-row |
| 138231 | TUN | SYR | CV (Manel) | 6-row |
| 138257 | LBN | SYR | CV (Litani) | 2-row |
| 138260 | AUS | SYR | CV (Keel) | 2-row |
| 138253 | LBY | SYR | CV (Katara) | 6-row |
| 138234 | IRQ | SYR | CV (IPA7) | 6-row |
| 138247 | EGY | SYR | CV (Giza 126) | 6-row |
| 138246 | EGY | SYR | CV (Giza 125) | 6-row |
| 138259 | SYR | SYR | CV (Furat3) | 2-row |
| 138249 | SYR | SYR | CV (Furat 2) | 2-row |
| 138248 | SYR | SYR | CV (Furat 1) | 6-row |
| 138233 | FRA | SYR | CV (Express) | 2-row |
| 138263 | ERI | SYR | CV (Demhay) | 2-row |
| 138243 | EGY | SYR | CV (CalM) | 6-row |
| 138239 | TUR | SYR | CV (Bulbul) | 2-row |
| 138261 | AUS | SYR | CV (Barque) | 2-row |
| 138254 | LBY | SYR | CV (Barjouj) | 6-row |
| 138264 | ERI | SYR | CV (Atsa) | 2-row |
| 138245 | GRC | SYR | CV (Aths) | 6-row |
| 138235 | MAR | SYR | CV (Arig8) | 6-row |
| 138215 | SYR | SYR | CV | 2-row |
| 138216 | SYR | SYR | CV | 2-row |
| 138219 | SYR | SYR | CV | 2-row |
| 138222 | SYR | SYR | CV | 2-row |
| 138225 | SYR | SYR | CV | 2-row |
| 138226 | SYR | SYR | CV | 6-row |
| 140405 | TJK | SYR | CV | 2-row |
| BMZ05-228 | UNK | SYR | CV | 2-row |
| BMZ05-229 | UNK | SYR | CV | 2-row |
| BMZ05-230 | UNK | SYR | CV | 6-row |
| BMZ05-231 | UNK | SYR | CV | 2-row |
| BMZ05-232 | UNK | SYR | CV | 2-row |
| BMZ05-233 | UNK | SYR | CV | 2-row |
| BMZ05-234 | UNK | SYR | CV | 2-row |
| BMZ05-235 | UNK | SYR | CV | 6-row |
| BMZ05-236 | UNK | SYR | CV | 2-row |
| BMZ05-237 | UNK | SYR | CV | 6-row |
| BMZ05-238 | UNK | SYR | CV | 6-row |
| BMZ05-239 | UNK | SYR | CV | 6-row |
| BMZ05-240 | UNK | SYR | CV | 2-row |
| BMZ05-241 | UNK | SYR | CV | 6-row |
| 138258 | UNK | SYR | Birlik | 2-row |
| 138256 | UNK | SYR | Batal-1 | 2-row |
| 138232 | UNK | SYR | Badia | 6-row |
| 138241 | UNK | SYR | Assala-04 | 6-row |
| 138227 | UNK | SYR | Alanda-01 | 6-row |

^a^Standarad code for country of origin, e.g. ALB=Albania, AFG=Afghanistan, AZE=Azerbaijan, BIH=Bosnia and Herzegovina, CHN=China, DEU=Deutschland, DZA= Algeria, EGY= Egypt, ETH= Ethiopia, GEO=Georgia, GRC=Greece, IND= India, IRN= Iran, IRQ= Iraq, JOR= Jordan, LBN= Lebanon, LBY= Libya, MAR= Morocco, OMN= Oman, PAK=Pakistan, PAL= Palestine, RUS= Russia, SAU=Saudi Arabia, SYR= Syria, SCG=Serbia and montenego, TJK= Tajikistan, TKM=Turkmenistan, TUN= Tunis, TUR=Turkey, UZB= Uzbekistan, YEM=Yemen, UNK= Unknown.
