# Supplementary material for: Allelic Variations of a Light Harvesting Chlorophyll A/B-Binding Protein Gene (Lhcb1) Associated with Agronomic Traits in Barley
Source: PLoS One. 2012 May 25;7(5):e37573. doi: 10.1371/journal.pone.0037573 (PMC3360778; doi:10.1371/journal.pone.0037573)
Supplement: Table S3 — Distribution of polymorphic SNPs across hyplotypes. SNPs relative to the most common sequence (haplotype H31) are indicated in boldface. The number of SNP positions is relative to the sequence on GenBank accession number AK359563.1. A horizontal dash indicates the absence of the indicated bases. (DOC) [file pone.0037573.s003.doc]

**Table S3** Distribution of polymorphic SNPs across hyplotypes. SNPs relative to the most common sequence (haplotype H31) are indicated in boldface. The number of SNP positions is relative to the sequence on GenBank accession number AK359563.1. A *horizontal dash* indicates the absence of the indicated bases.

| Haplotypes | SNP position | | | | | | | | | | | | | | | | | | | | | | | Total |
| --- | --- | --- | --- | --- | --- | --- | --- | --- | --- | --- | --- | --- | --- | --- | --- | --- | --- | --- | --- | --- | --- | --- | --- | --- |
|  | 81 | 132 | 220 | 252 | 276 | 334 | 411 | 463 | 490 | 531 | 534 | 550 | 572 | 589 | 669 | 781 | 805 | 907 | 961 | 1006 | 1049 | 1054 | 1060 |  |
| H1 | G | G | C | C | G | G | A | G | T | C | **T** | C | T | G | C | G | G | C | T | G | GTGC | CTGCT | A | 1 |
| H2 | **C** | G | C | C | G | G | A | G | T | C | C | C | T | G | C | **A** | G | C | T | G | GTGC | CTGCT | A | 1 |
| H3 | G | G | C | C | G | G | A | G | T | C | C | C | **C** | G | C | **A** | G | C | T | G | GTGC | CTGCT | A | 1 |
| H4 | G | G | C | C | **A** | G | A | G | T | C | C | C | **C** | G | C | G | G | C | T | G | — | — | — | 1 |
| H5 | G | G | C | C | G | G | A | G | T | C | C | C | T | G | C | G | G | C | T | G | — | — | — | 1 |
| H6 | **C** | G | C | C | G | G | A | G | T | C | C | C | T | G | C | G | G | **A** | T | G | GTGC | CTGCT | A | 1 |
| H7 | **C** | G | C | C | G | G | A | G | T | C | C | C | T | G | C | G | G | C | T | G | GTGC | CTGCT | A | 1 |
| H8 | G | G | C | C | G | G | A | G | T | **T** | C | C | T | G | C | G | G | C | T | C | GTGC | CTGCT | A | 1 |
| H9 | G | G | C | C | G | G | A | G | T | **T** | C | C | T | G | C | G | G | C | T | G | GTGC | CTGCT | A | 1 |
| H10 | G | G | C | C | G | G | A | G | T | C | C | T | T | G | **T** | G | **A** | C | T | G | GTGC | CTGCT | A | 1 |
| H11 | G | G | C | C | G | G | A | G | T | C | C | **T** | T | G | C | G | G | C | T | C | GTGC | CTGCT | A | 1 |
| H12 | G | G | C | **T** | G | G | A | G | T | C | C | C | T | G | C | G | G | **A** | T | G | GTGC | CTGCT | A | 1 |
| H13 | G | G | C | C | G | G | **G** | G | T | C | C | C | T | **A** | C | G | G | **A** | T | G | GTGC | CTGCT | A | 1 |
| H14 | G | G | C | C | **A** | G | A | **A** | T | C | C | C | T | G | C | G | G | C | T | **C** | GTGC | CTGCT | A | 1 |
| H15 | G | G | **A** | **T** | G | G | A | G | T | C | C | C | T | G | C | G | G | C | T | G | GTGC | CTGCT | A | 2 |
| H16 | G | G | C | C | G | **A** | A | G | T | C | C | C | T | G | C | G | G | C | T | G | GTGC | CTGCT | A | 2 |
| H17 | G | G | C | C | G | G | A | G | T | C | C | **T** | T | G | C | G | G | C | T | G | GTGC | CTGCT | A | 2 |
| H18 | G | G | C | C | G | G | A | G | T | C | C | C | **C** | G | C | G | G | C | T | G | GTGC | CTGCT | A | 2 |
| H19 | G | **T** | C | C | **A** | G | A | G | **C** | C | C | C | **C** | G | C | G | G | C | T | G | GTGC | CTGCT | A | 3 |
| H20 | G | G | C | C | G | G | A | G | T | C | C | C | T | G | C | G | G | C | **C** | G | GTGC | CTGCT | A | 3 |
| H21 | G | G | C | C | G | G | A | G | T | C | C | C | T | G | C | G | G | C | T | **C** | GTGC | CTGCT | A | 3 |
| H22 | G | G | C | C | G | G | A | **A** | T | C | C | C | T | G | C | G | G | C | T | G | GTGC | CTGCT | A | 3 |
| H23 | G | G | C | C | G | G | **G** | G | T | C | C | C | T | **A** | C | G | G | C | T | G | GTGC | CTGCT | A | 5 |
| H24 | G | G | C | **T** | G | G | A | G | T | C | C | C | T | G | C | G | G | C | T | G | GTGC | CTGCT | A | 6 |
| H25 | G | G | C | C | **A** | G | A | G | T | C | C | C | **C** | G | C | G | G | **A** | T | G | GTGC | CTGCT | A | 6 |
| H26 | G | G | C | C | G | G | G | G | T | C | C | C | T | A | C | G | G | C | **C** | G | GTGC | CTGCT | A | 9 |
| H27 | G | G | C | C | G | G | A | **A** | T | C | C | C | T | G | C | G | G | C | T | C | GTGC | CTGCT | A | 14 |
| H28 | G | G | C | C | **A** | G | A | G | T | C | C | C | **C** | G | **T** | G | **A** | C | T | G | GTGC | CTGCT | A | 16 |
| H29 | G | G | C | C | **A** | G | A | G | T | C | C | C | **C** | G | C | G | G | C | T | G | GTGC | CTGCT | A | 41 |
| H30 | G | G | C | C | G | G | A | G | T | C | C | C | T | G | C | G | G | **A** | T | G | GTGC | CTGCT | A | 68 |
| H31 | G | G | C | C | G | G | A | G | T | C | C | C | T | G | C | G | G | C | T | G | GTGC | CTGCT | A | 93 |
